# Induction of Transforming Growth Factor Beta Receptors following Focal Ischemia in the Rat Brain

**DOI:** 10.1371/journal.pone.0106544

**Published:** 2014-09-05

**Authors:** Gabriella Pál, Gábor Lovas, Arpád Dobolyi

**Affiliations:** 1 Laboratory of Neuromorphology, Department of Anatomy, Histology and Embryology, Semmelweis University, Budapest, Hungary; 2 Department of Neurology, Semmelweis University, Budapest, Hungary; 3 Department of Neurology, Jahn Ferenc Teaching Hospital, Budapest, Hungary; 4 Laboratory of Molecular and Systems Neurobiology, Institute of Biology, Hungarian Academy of Sciences and Eötvös Loránd University, Budapest, Hungary; National University of Singapore, Singapore

## Abstract

Transforming growth factor-βs (TGF-βs) regulate cellular proliferation, differentiation, and survival. TGF-βs bind to type I (TGF-βRI) and II receptors (TGF-βRII), which are transmembrane kinase receptors, and an accessory type III receptor (TGF-βRIII). TGF-β may utilize another type I receptor, activin-like kinase receptor (Alk1). TGF-β is neuroprotective in the middle cerebral artery occlusion (MCAO) model of stroke. Recently, we reported the expression pattern of TGF-β1-3 after MCAO. To establish how TGF-βs exert their actions following MCAO, the present study describes the induction of TGF-βRI, RII, RIII and Alk1 at 24 h, 72 h and 1 mo after transient 1 h MCAO as well as following 24 h permanent MCAO using in situ hybridization histochemistry. In intact brain, only TGF-βRI had significant expression: neurons in cortical layer IV contained TGF-βRI. At 24 h after the occlusion, no TGF-β receptors showed induction. At 72 h following MCAO, all four types of TGF-β receptors were induced in the infarct area, while TGF-βRI and RII also appeared in the penumbra. Most cells with elevated TGF-βRI mRNA levels were microglia. TGF-βRII co-localized with both microglial and endothelial markers while TGF-βRIII and Alk1 were present predominantly in endothels. All four TGF-β receptors were induced within the lesion 1 mo after the occlusion. In particular, TGF-βRIII was further induced as compared to 72 h after MCAO. At this time point, TGF-βRIII signal was predominantly not associated with blood vessels suggesting its microglial location. These data suggest that TGF-β receptors are induced after MCAO in a timely and spatially regulated fashion. TGF-β receptor expression is preceded by increased TGF-β expression. TGF-βRI and RII are likely to be co-expressed in microglial cells while Alk1, TGF-βRII, and RIII in endothels within the infarct where TGF-β1 may be their ligand. At later time points, TGF-βRIII may also appear in glial cells to potentially affect signal transduction via TGF-βRI and RII.

## Introduction

Receptors of the transforming growth factor-beta (TGF-β) superfamily are receptor serine kinases [1], [2]. Their canonical signal transduction pathway includes SMAD proteins, which need to be phosphorylated to exert their effects within the nucleus [3]. Based on their role and sequence homology, the receptors of the TGF-β superfamily can be divided as type I receptors, which can phosphorylate SMAD proteins, type II receptors, required for the action of type I receptors, and accessory receptors that may play a role in recruiting the ligands [4]. TGF-β receptor I (TGF-β RI), or activin-like receptor kinase 5, was initially recognized as the receptor of all 3 types of TGF-βs, TGF-β1, -β2, and -β3, which are all dimeric ligands [5], [6]. The ligands bind to TGF-β receptor II (TGF-β RII), which in turn forms the functional receptor with TGF-β RI and phosphorylates it [7]. A functional receptor is a heterotetramer consisting of 2 TGF-β RI, and 2 TGF-β RII [8]. In addition, TGF-β receptor III (TGF-β RIII), or betaglycan, has the ability to influence the TGF-β receptor complex formed by TGF-β RI and RII [9]. Although TGF-β RIII has a short intracellular domain, it presents TGF-βs to TGF-β RII [10], [11] and may bind some other ligands including inhibin [12]. TGF-β receptor III is particularly important for the recognition of TGF-β2, which binds poorly to the TGF-β receptor [13]. More recently, another type I receptor, activin-like receptor kinase 1 (Alk1) was shown to signal TGF-β1, and -β3 in addition to bone morphogenic protein 9 [14]. It has also been demonstrated that the actions through Alk1 are often different from, sometimes even antagonistic to those exerted by TGF-β RI [15].

The knowledge available on the TGF-βs and their receptors in the brain is also rapidly accumulating. Immunohistochemical studies suggested that in the intact brain, TGF-β1 is present only in choroid plexus epithelial and meningeal cells while the distribution of TGF-β2 and -β3 are more widespread [16], [17]. In turn, TGF-β1 is induced in response to injury including the focal ischemia elicited by middle cerebral artery occlusion (MCAO), an animal model of stroke [18], [19]. We recently demonstrated by using in situ hybridization histochemistry that TGF-β2 and -β3 can also be induced by MCAO albeit with a different temporal and spatial pattern. While TGF-β1 appears first in the penumbra and later within the infarct in microglial cells, TGF-β2 is induced in neurons in the penumbra and in the intact ipsilateral cerebral cortex away from the lesion [20]. TGF-βs were shown to be neuroprotective following MCAO [21]. In addition to neuroprotection, TGF-βs may also participate in regenerative processes as they increase adult [22] and promote neuronal cell fate of cortical and hippocampal progenitors [23]. TGF-β1 administered into the brain reduced the infarct size in experimental models of ischemia [24]–[26] while antagonizing the endogenous action of TGF-β1 with the injection of a soluble TGF-β type II receptor, which binds TGF-β1 and prevents its biological actions, resulted in a dramatic increase in infarct area [27]. Recently, small molecule inhibitors of TGF-β signaling became available and are used in clinical trials [28]. Neuroprotective actions of some of these novel drugs have also been suggested [29]. However, it remains to be established which receptors are involved in these actions. A study using RNase protection assays demonstrated the presence of mRNA of TGF-β RI, RII, and RIII in different brain regions [30]. Immunohistochemical studies reported that TGF-β RI and RII are present in the intact brain both in neurons and glial cells [17], [31]. Furthermore, induction in the levels of TGF-β RI and RII immunoreactivities were found in response to focal [32], [33], and global ischemia [34]. TGF-β RIII was also found in the brain, with abundance in reproductive regions [35]. In contrast, Alk1 was generally confined to endothelial cells [36] with an increased expression by vascular injury [37]. In addition, Alk1 may also be present in neurons [38]. The neuroprotective functions of TGF-βs suggest the involvement of TGF-β receptors in the tissue response to focal hypoxia. Such involvement may include gene expressional changes whose identification will contribute to our understanding of the mechanisms of actions by TGF-βs. Therefore, we addressed the following questions in the present study: 1. How are mRNAs of TGF-β RI, RII, RIII, and Alk1 distributed following 1 h MCAO and permanent 24 h MCAO in the rat brain? 2. What is the time course of induction of mRNA of TGF-β RI, RII, RIII, and Alk1 following MCAO? 3. Which cell types express the different subtypes of TGF-β receptor following MCAO? *In situ* hybridization histochemistry, which we used previously to describe the distribution of TGF-βs in response to MCAO [20] and its combination with immunolabeling for neuronal (NeuN), astrocyte (S100), microglial (ionized calcium-binding adapter molecule 1 - Iba1), endothelial cells (von Willebrand factor – vWF), and smooth muscle cells (alpha smooth muscle actin – alpha-SMA) markers were applied to identify the cells that express the different TGF-β receptors in the ischemic rat brain.

## Materials and Methods

### Animals

The Animal Examination Ethical Council of the Animal Protection Advisory Board at Semmelweis University, Budapest specifically approved this study (Permit Number: 2268-4/2012). Thus, the procedures involving rats were carried out according to experimental protocols that meet the guidelines of the Animal Hygiene and Food Control Department, Ministry of Agriculture, Hungary, which is in accordance with EU Directive 2010/63/EU for animal experiments. A total of 42 adult, male Wistar rats (300–450 g body weight; Charles Rivers Laboratories, Hungary) were used in this study. All efforts were made to minimize the number of animals used, and also their suffering. Animals were kept on standard laboratory conditions with 12-h light, 12-h dark periods (lights on at 6.00 a.m.), and supplied with dry rat food and drinking water *ad libitum*. Rats were kept 3 per cage at a temperature of 22±1°C. Rats were anaesthetized with an intramuscular injection (0.2 ml/300 g body weight) of anesthetics containing ketamine (60 mg/ml) and xylazine (8 mg/ml) for middle cerebral artery occlusion, transcardial perfusion, or decapitation.

### Middle cerebral artery occlusion

Focal ischemia was induced using a modified intraluminal suture method of the described previously [39]. Briefly, left common, internal and external carotid arteries were exposed through a midline neck incision and were carefully dissected from the surrounding tissues under an operating microscope. After electrocoagulation of the external and common carotid arteries, a 3–0 silicon rubber-coated monofilament (Doccol, Redlands, CA) was inserted through the common carotid artery into the internal carotid artery 18 to 20 mm beyond the carotid bifurcation to the base of the middle cerebral artery. The pterygopalatine branch of the internal carotid artery was exposed before the insertion in order to avoid the filament turning into it. An atraumatic aneurysm clip (Codman, Johnson and Johnson, Le Locle, NE, Switzerland) was placed on the internal carotid artery to prevent bleeding. The clip and the monofilament were removed 1 h later (except for 24 h permanent MCAO), and the incision was sutured. The rats were sacrificed 24 h, 72 h, or 1 month after the beginning of the reperfusion and their brains were dissected and cut coronally at the level of the bregma. The anterior parts of the brains were stained with 2, 3, 5-triphenyltetrazolium chloride (TTC) and the posterior parts frozen for *in situ* hybridization histochemistry.

### Allocation of operated animals into experimental groups

Six rats were used in each of the following 4 groups: (a) dissection at 24 h after 1 h MCAO, (b) rats with a permanent MCAO dissected 24 h after the occlusion, dissection at (c) 72 h, and (d) 1 month after 1 h MCAO. Some additional animals were perfused for immunohistochemistry. The number of perfused animals included 4-4 rats sacrificed 24 h, 72 h, and 1 month after 1 h MCAO. A further 6 sham-operated rats were used (2 for each time points, one freshly dissected and one perfused).

### Probe preparation

Cerebral cortex was dissected from a fresh brain, quickly frozen on dry ice, kept at −80°C before total mRNA was isolated using Trizol Reagent (Invitrogen, Carlsbad, CA) according to the manufacturer's instructions. After diluting total RNA to 2 µg/µl, RNA was treated with Amplification Grade DNase I (Invitrogen), and cDNA was synthesized with a Superscript II reverse transcriptase kit (Invitrogen) according to the manufacturer's instructions. After tenfold dilution, 2.5 µl of the resulting cDNA was used as template in PCRs performed with iTaq DNA polymerase (Bio-Rad Laboratories, Hercules, CA) in total volumes of 12.5 µl under the following conditions: 95°C for 3 minutes, followed by 35 cycles of 95°C for 0.5 minutes, 60°C for 0.5 minutes, and 72°C for 1 minute. Primers were used at 300 nM final concentration for TGF-β RI (primer pair A: CAATTGCAAGGACCATTGTG and ATGTGAAGATGGGCAAGACC, B: ATCTTGGGAAGGGCAGAGTT and CACCAGTGAGGAGACCCAAT), TGF-β RII (primer pair A: GTGGAAAACGGAGAAGGACA and AGCTCTTGAGGTCCCTGTGA, B: GTGTGACTTCGGGTTGTCCT and TTTCATGCTCTCCACACAGG), TGF-β RIII (primer pair A: GGCTTGAGAACAACGAGGAG and TCCCTGAGTAGCCATTGGTC, B: TTTGTCCAGGTGTCCAAACA and GGCACTTTTGGAGTTGGTGT), and Alk1 (primer pair A: AGCGATTACCTGGACATTGG and GTACCAGCACTCTCGCATCA, B: TTTCAGCAGTGTGCAAGGAC and CATTTGGAGAATGCCACCTT). The calculated lengths of the PCR products are 355 and 252 base pairs (bp) for TGF-β RI (663–1017 and 2789–3040 bp of GenBank accession No. NM_012775.2), 290 and 280 bp for TGF-β RII (1109–1398 and 1436–1715 bp of GenBank accession No. NM_031132.3), 366 and 331 bp for TGF-β RIII (1404–1769 and 3178–3508 bp of GenBank accession No. NM_017256.1), and 345 and 306 bp for Alk1 (1219–1563 and 3096–3401 bp of GenBank accession No. NM_022441.2). The primers were chosen to generate probes that do not overlap but recognize all known RNA species for the particular gene. PCR products were run on gel, and pictures were taken with a digital camera. Then, the PCR products were purified from gel, inserted into TOPO TA cloning vectors (Invitrogen), and transformed chemically into competent bacteria according to the manufacturer's instructions. Plasmids were purified from five to seven colonies and applied as templates in PCRs with specific primer pairs to select plasmids containing specific inserts. A positive plasmid for each probe was applied as template in PCRs, using primer pairs specific for the probe and also containing T7 RNA polymerase recognition site (GTAATACGACTCACTATAGGGCGAATTGGGTA) added to the reverse primers and T3 RNA polymerase recognition site (AATTAACCCTCACTAAAGGGAACAAAAGCTGG) added to the forward primers. At the end, the identities of the cDNA probes were verified by sequencing them with T7 primers.

### *In situ* hybridization histochemistry

Brains of 27 rats (6 in all 4 groups and 1-1 sham operated) were removed and the fresh tissue was quickly frozen on dry ice, and kept at −80°C. Serial coronal sections (12 µm thick) were cut using a cryostat from bregma level +4 mm to −6 mm [40], mounted on positively charged slides (SuperfrostPlus, Fisher Scientific, Pittsburgh, PA), dried, and stored at −80°C until use. The brain sections were collected in such a way that consecutive sections were mounted on 18 parallel slides. For *in situ* hybridization, [^35^S]UTP-labeled riboprobes were generated from the DNA probes containing T7 and T3 RNA polymerase recognition sites using a MAXIscript transcription kit (Ambion, Austin, TX). Antisense riboprobes were prepared using T7 RNA polymerase while sense control probes were prepared using T3 RNA polymerase. The preparation of tissue was performed using mRNAlocator Kit (Ambion), according to the manufacturer's instructions. For hybridization, we used 80 µl hybridization buffer and labeled probes of 1 million DPM activity per slide. Tissue was prepared using an mRNA-locator Kit (Ambion) according to manufacturer's instructions. For hybridization, we used 80 µl hybridization buffer and 1 million DPM of labeled probe per slide. Washing procedures included a 30 min incubation in RNase A, followed by decreasing concentrations of sodium-citrate buffer (pH = 7.4) at room temperature, and then at 65°C. After drying, slides were dipped in NTB nuclear track emulsion (Eastman Kodak, Rochester, NY), stored for 3 weeks at 4°C for autoradiography, developed with Kodak Dektol developer, fixed with Kodak fixer, counterstained with Giemsa, and coverslipped with Cytoseal 60 (Stephens Scientific, Riverdale, NJ, USA).

### Quantitation of in situ hybridization histochemistry: densitometric and statistical analysis

Sections of 6-6 brains from the following groups of animals were included in the analysis: rats 24 h following 1 h MCAO, rats with a permanently occluded middle cerebral artery at 24 h following MCAO, rats at 72 h and 1 mo following 1 h MCAO. Dark-field photomicrographs were taken of the sections at 3-3 positions from within the lesions, the penumbra and the adjacent intact tissue outside of the lesion using a 40× objective. The density of autoradiography grains within 400×400 µm areas (0.16 mm^2^) of coronal brain sections was counted, the averages of the 3 positions were considered as one value. The pixel number of white area (lighter than an arbitrary grayness used for all the images) was calculated for the images using ImageJ 1.47v (National Institutes of Health, USA) software. The values were used to quantify the mRNA levels. The mRNA levels at the different time points were compared using one-way ANOVA. The mRNA levels at different locations and time points were compared using two-way ANOVA. The individual values were compared using Bonferroni's multiple comparison post-hoc tests.

### Cresyl-violet staining

Alternative series of sections from the MCAO treated rats were mounted consecutively on gelatin-coated slides and dried. Sections were stained in 0.1% cresyl-violet dissolved in PB, then differentiated in 96% ethanol containing acetic acid. Sections were then dehydrated and coverslipped with Cytoseal 60 (Stephens Scientific).

### Quantitation of the lesioned brain areas

A section with the largest apparent lesion was selected from each brain. Its antero-posterior bregma coordinate was always between +0.8 and −0.8 mm. These sections were labeled with cresyl-violet (Nissl) staining as described above. Images of Nissl-stained brain sections from each rat were obtained and areas of the infarctions and the ipsilateral hemispheres were manually demarcated followed my measurement of the areas using ImageJ 1.47v (National Institutes of Health, USA). The percentage of the infarct area to the ipsilateral hemisphere was calculated.

### Tissue collection for immunolabeling

Rats (n = 15, 4 at each time point and 3 sham operated) were deeply anesthetized and perfused transcardially with 150 ml saline followed by 300 ml 4% paraformaldehyde prepared in phosphate buffer (PB; pH = 7.4). Brains were removed and postfixed in 4% paraformaldehyde for 24 h and then transferred to PB containing 20% sucrose for 2 days. Serial coronal brain sections were cut at 20 µm on a cryostat between 4.0 and −7.0 mm bregma levels. Subsequently, the sections were processed for immunohistochemistry or for a combination of *in situ* hybridization and immunohistochemistry. The brain sections were collected in such a way that consecutive sections were mounted on 18 parallel slides.

### Immunohistochemistry

Slide-attached sections were pretreated with 3% hydrogen peroxide for 10 min followed by 1% bovine serum albumin in PB containing 0.5% Triton X-100 for 30 min at room temperature. Then, parallel series of sections were placed in one of the following primary antisera for 24 h at room temperature: mouse anti-NeuN as a marker of neuronal nuclei (1∶500; Millipore, Billerica, MA, cat. number: MAB377), mouse anti-S-100, as a marker of astrocytes (1∶5000 Sigma-Aldrich, cat. number: S2532), rabbit anti-ionized calcium-binding adapter molecule 1 (Iba1) as a marker of microglial cells (1∶1000; Wako, cat. number: 019–197419), mouse anti-alpha smooth muscle actin (alpha-SMA) as a marker of smooth muscle cells of blood vessels (1∶2000 Abcam, cat. number: ab7817), rabbit anti-Von Willebrand factor (vWF) as an endothelial marker (1∶1500 Abcam, cat. number: ab6994). The specificity of the antibodies used in the present study has been validated previously by the companies using western blotting. In addition, our own results also argue for specific labeling based on the morphology of the labeled cells. After incubation in primary antisera, the sections were washed, and incubated in either biotinylated anti-rabbit secondary antibody (1∶1000; Vector Laboratories, Burlingame, CA, USA) or anti-mouse secondary antibody (1∶1000; Jackson ImmunoResearch, West Grove, PA), for 1 h followed by washes and incubation in avidin-biotin-horseradish peroxidase complex (1∶500; Vector Laboratories) for 1 h. Finally, the sections were treated with 0.06% DAB and 0.003% H_2_O_2_ in Tris hydrochloride buffer (0.05 M, pH = 8.2) for 10 min, and coverslipped with Cytoseal 60 (Stephens Scientific).

### Combination of immunohistochemistry and in situ hybridization histochemistry

Slide attached sections of perfused brains were first processed for in situ hybridization, as described above. Thus, tightly bound RNA-RNA pairs were already formed by the time immunohistochemistry was performed, immediately before dipping the slides into autoradiographic emulsion. In addition, the solutions used for perfusion and immunohistochemistry were prepared with DAPC-treated RNAse-free water, which ensured that the labeling intensity of the in situ hybridization histochemistry did not decrease significantly. The immunolabeling protocol was the same as that used for single labeling immunohistochemistry. Immunoreactivity was visualized using DAB reactions, after which the in situ hybridization procedure was continued by dipping the slides into emulsion. Each double labeling experiment included controls, which went through the double labeling procedure without application of radioactive in situ hybridization probes. These controls demonstrated that the DAB signal did not induce an autoradiography signal.

### Quantitative analysis of double labeling experiments

A cell was considered to express TGF-β receptor if the number of autoradiography grains accumulated in a seemingly Gaussian distribution around a center was at least 3 times higher than the background level in an area corresponding to an average cell size (a circle with a diameter of 25 µm). The background typically contained 3–4 grains, but always less than 7 grains, per cell in the in situ hybridization histochemistry. A TGF-β receptor-expressing cell was considered immunopositive if at least half of the area of the circle containing the accumulation of autoradiography grains contained immunoreactivity for a particular marker. For quantitation, we counted the number of TGF-β receptor-expressing cells and the number of double-labeled cells defined above (TGF-β Receptor + NeuN, S-100, Iba1 or vWF) in 400×400 µm rectangular areas ipsilateral but away from the lesion, in the penumbral and the lesioned cerebral cortex.

### Histological analysis and image processing

The sections were examined using an Olympus BX60 light microscope in both dark-field and bright-field. Images were captured at 2048×2048 pixel resolution with a SPOT Xplorer digital CCD camera (Diagnostic Instruments, Sterling Heights, MI) using a 4× objective for dark-field images, and 4–40× objectives for bright-field images. The contrast and sharpness of the images were adjusted using the “levels” and “sharpness” commands in Adobe Photoshop CS 8.0. Full resolution was maintained until the photomicrographs were printed, at which point the images were adjusted to a resolution of 300 dpi.

## Results

**Figure 1:**
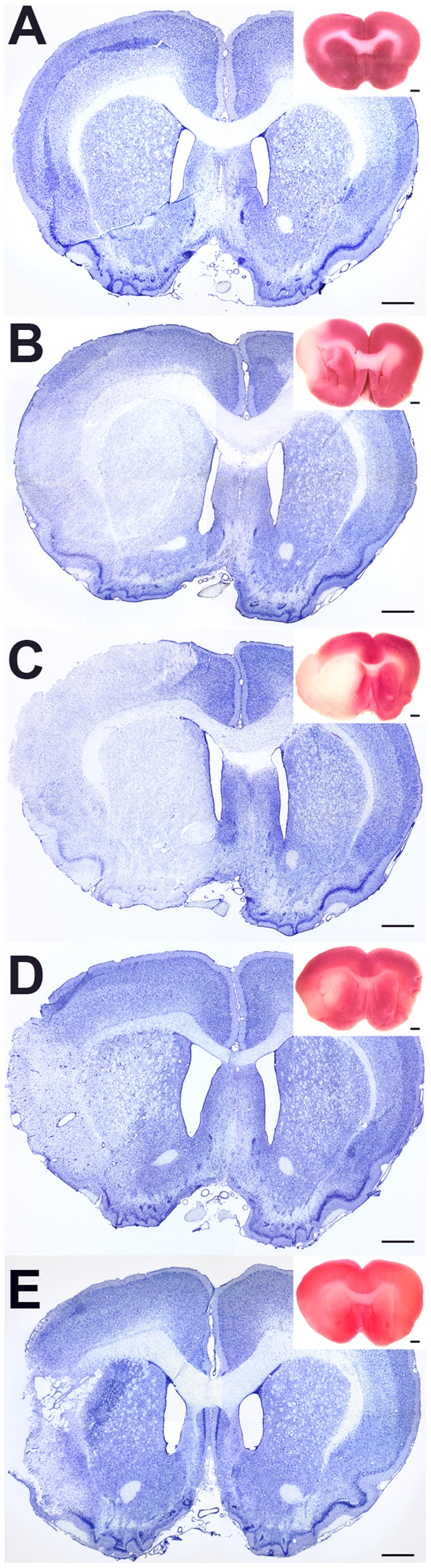
Nissl and TTC staining of the lesioned brain at different time points after focal ischemia. Coronal sections demonstrate the damage caused by the experimental manipulations. The lesion can be seen as the area with lighter appearance in the Nissl and TTC sections. The Nissl and the TTC stained sections are at different rostrocaudal levels because TTC labeling was applied to the frontal part of the brains while Nissl staining was performed on sections intermingled with those used for in situ hybridization histochemistry at a level where the size of the lesion was maximal. A: Sham operated; there is no sign of lesion. B: 24 hours after transient MCAO; the infract area is visible in the striatum and the cerebral cortex. C: 24 hours after permanent MCAO; the lesion is more pronounced than following transient MCAO. D: 72 hours following transient MCAO; the mass of invading cells are visible. E: 1 month after MCAO; some tissue disappears, while other parts of the infarct area are completely invaded by non-neuronal cells. Scale bars  = 1 mm.

The basal expression level of TGF-β receptors was very low and not topographically organized in the brain except for TGF-β RI, which was abundant in the cerebral cortex, most peculiarly in layer IV (Fig. S1). Sham operations did not induce any TGF-β receptor mRNA or altered the pattern of basal expressions even in brain areas supplied by the middle cerebral artery (Fig. S1). The 1 h MCAO we performed resulted in large lesions that included large parts of the ipsilateral striatum and a considerable part of the cortex. The lesion was visible using TTC staining as the infarct did not show the red labeling characteristic of live tissue in freshly dissected brains (Fig. 1). TTC staining indicated successful lesion for each brain involved in the study. Nissl staining of the sections also demonstrated the extent of the lesion (Fig. S2). Moreover, Nissl staining provided valuable information on the size of the infarct area. The size of the lesion following 24 h after MCAO was 46.3±2.9% of the ipsilateral hemisphere measured in freshly dissected brains. The size of the lesion was larger following permanent than 1 h transient MCAO, the lesioned area accounted for 59.9±5.1% of the ipsilateral hemisphere. At 72 h, and even more so at 1 mo following MCAO, the apparent size of the lesion was reduced as compared to 24 h after MCAO (47.7±4.7% at 72 h and 35.3±2.8% at 1 month). It was also observed at these time points that invading cells occupied the lesioned neural tissue(Fig. S2).

We describe that mRNA of each type of TGF-β receptor showed a specific topographical expression pattern in response to focal ischemia ipsilateral to the lesion. In contrast, we never observed alterations in mRNA expression in the contralateral side of the brain (Fig. S1). However, the pattern of induction on the lesion side changed with time after MCAO. The cell types expressing the different types of TGF-β receptors were also identified by co-localization with known cellular markers.

### The expression patterns of TGF-β mRNAs in the brain following MCAO

#### 24 h after transient MCAO

**Figure 2:**
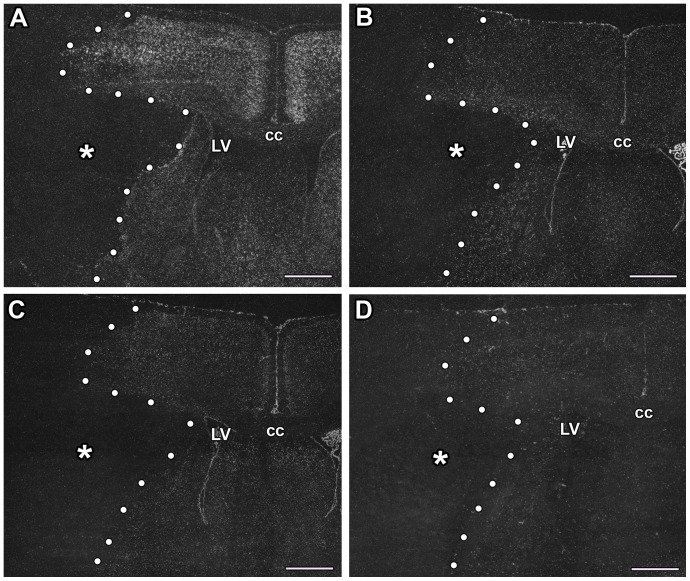
The expression of TGF-β receptors at 24 h following MCAO. Dark-field photomicrographs of sections labeled by in situ hybridization histochemistry show the mRNA of TGF-β receptors. The lesion sites are indicated by star symbols (*) and the borders of lesions are demarcated by white dots. Away from the lesion, a low level of TGF-β RI expression is seen in the caudate putamen and a higher level in the cortex (A). The basal expression level of TGF-β RII (B), TGF-β RIII (C), and ALK1 mRNA (D) is very low. Abbreviations: cc - corpus callosum, LV - lateral ventricle. Scale bars  = 1 mm.

TGF-β receptors were not present within the infarct area, in fact, even their very low level of expression disappeared as demonstrated by in situ hybridization histochemistry (Fig. 2). The expression did not change outside of the lesion. Thus, it was similar to their expression in the contralateral side and in the sham operated controls. A low level of TGF-β RI expression was observed in the intact caudate putamen and a higher level in the cortex while the basal expression level of TGF-β RII, RIII, and ALK1 mRNA was very low. The peri-infarct area represented a transition between the lesion and the intact tissue without specific induction in the mRNA level of any TGF-β receptor (Fig. 2). Even though permanent MCAO led to larger lesion, the expression patters of TGF-β receptors was similar to transient lesions: there were no visible inductions in the mRNA levels of TGF-β receptors in any brain region.

#### 72 h after transient MCAO

**Figure 3:**
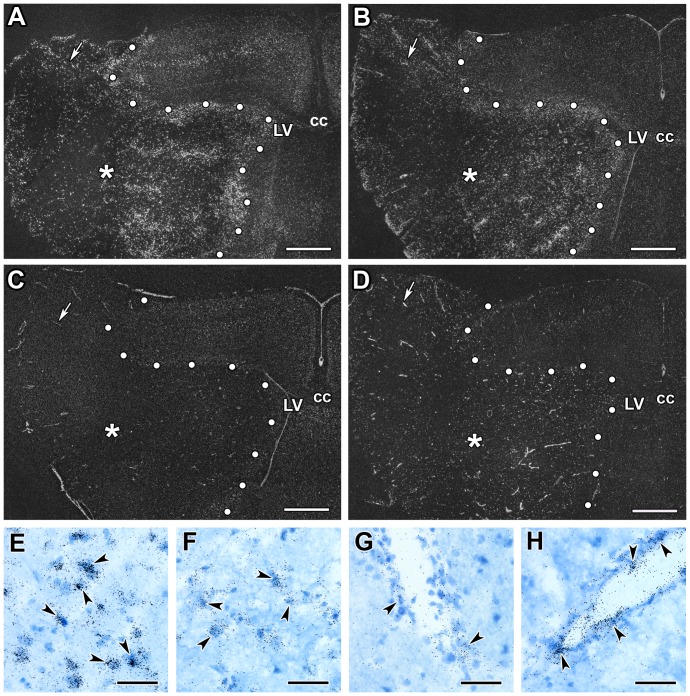
The induction of TGF-β receptors at 72 h following MCAO. Dark- and bright-field images demonstrate the induction of mRNA of the TGF-β receptors. The lesion sites are indicated by star symbols (*) and the borders of lesions are demarcated by white dots. A: TGF-β RI expression is induced in the penumbra immediately outside the lesion as well as within the infarct area. B: The mRNA of TGF-β RII is also induced in the penumbra and also within the lesion. C: TGF-β RIII mRNA expressing cells were present within the lesioned area with appearance suggesting expression in blood vessels. D: ALK1 was also seen to be induced around the vessels within the infarct area. The fields indicated by the white arrows in A–D is enlarged and shown in bright field in E–H. Cells expressing TGF-β RI (E), TGF-β RII (F), TGF-β RIII (G), and Alk1 (H) are indicated by black arrowheads. The mRNA of TGF-β RIII and Alk1 are around blood vessels. Abbreviations: cc - corpus callosum, LV - lateral ventricle. Scale bars: 1 mm for A-D, and 50 µm for E–H.

**Figure 4:**
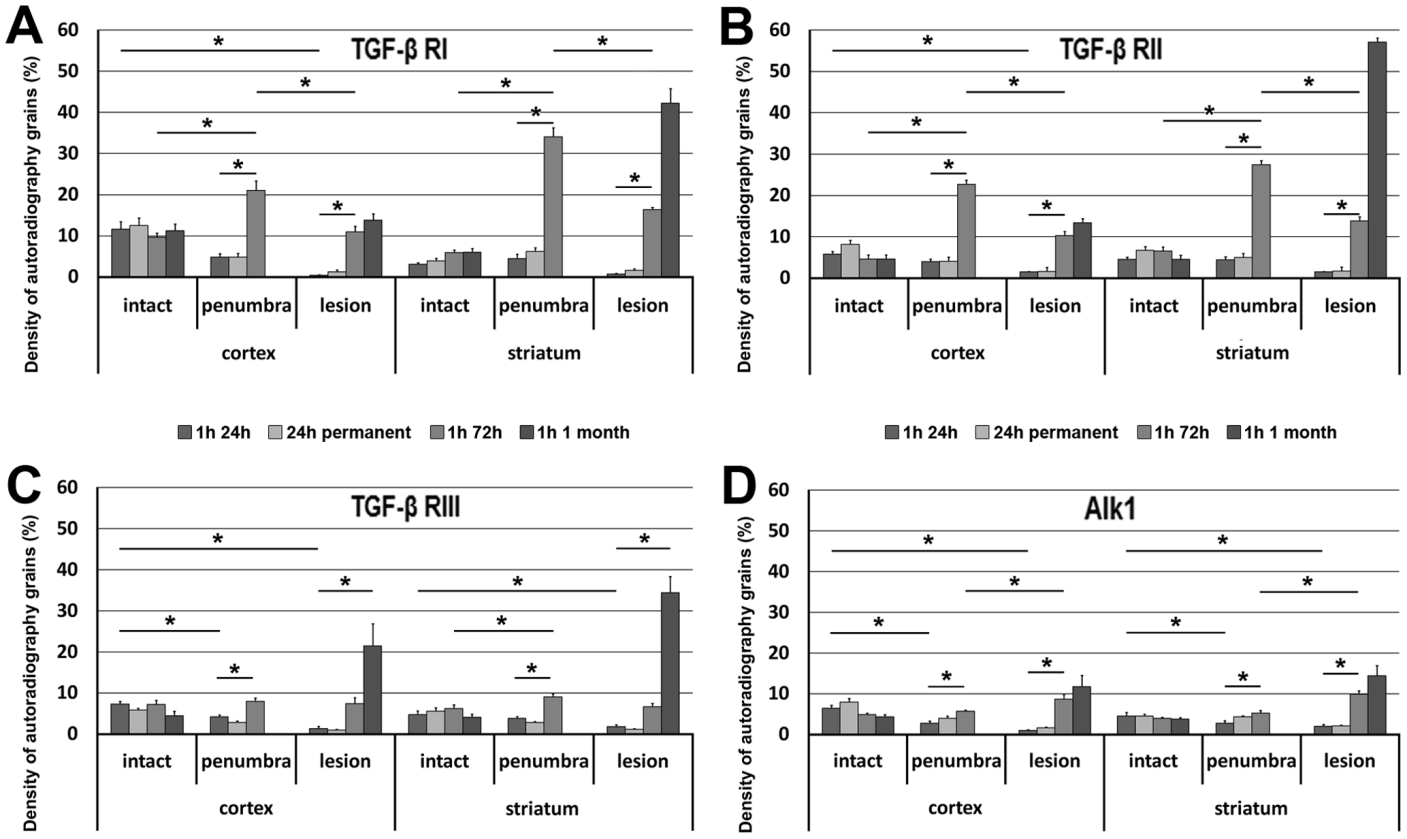
Alterations in the mRNA levels of receptors of TGF-βs following MCAO. The density of autoradiography grains (Y axis) was calculated for the TGF-β RI (A), the TGF-β RII (B), the TGF-β RIII (C), and Alk1 (D). Analysis was performed in the cortex and the striatum in 4 cases: 24 h after transient (1 h) and permanent MCAO, 72 h, and 1 month after transient MCAO. The penumbra was not examined 1 month after MCAO as it was not identifiable any more. The star symbol (*) indicates significantly (p<0.05) different values.

The most dramatic change at this time point was that TGF-β RI mRNA appeared within the lesion. Some cells were strongly labeled with TGF-β RI mRNA. Their distribution was uneven. However, they were present generally all over in the infarct area (Fig. 3A). In addition, TGF-β RI-expressing cells also appeared around the lesion in all cerebral layers as well as in the caudate putamen (striatum). Thus, the density of TGF-β RI mRNA was higher in the penumbra than in the intact brain tissue (2-way ANOVA for cortex and Bonferroni posttest for 72 h: F = 32.99, t = 6.501, p<0.001; 2-way ANOVA for striatum and Bonferroni posttest for 72 h: F = 441.6, t = 17.18, p<0.001; Fig. 4A). An induction of TGF-β RI mRNA was not found in the intact tissue outside of the lesion including ipsilateral and contralateral hemispheres.

TGF-β RII mRNA was also induced at 72 h following MCAO (Fig. 3B). In fact, the distribution of TGF-β RII expression was similar to that of TGF-β RI except in the intact cerebral cortex, where TGF-β RII showed the same, very low level of expression as in the intact brain (Fig. 4B). High magnification bright-field images demonstrated that the in situ hybridization signal, the autoradiography grains were distributed above cell bodies (Fig. 3E, F). In bright-field, it was also apparent that the signal has lower intensity for TGF-β RII than for TGF-β RI (Fig. 3E, F).

TGF-β RIII mRNA showed an elevated level within the lesion and in the penumbra (1-way ANOVA for cortex and Bonferroni posttest: F = 19.29, t = 4.40; p<0.01; for striatum: F = 42.09, t = 7.12; p<0.001; Fig. 3C, 4C). The increase was less pronounced than for other TGF-β receptors but still clearly visible. The labeled cells were distributed in large areas. It was also obvious using bright-field analysis of Giemsa counterstained sections that TGF-β RIII mRNA was associated with blood vessels (Fig. 3G) within the infarct and the penumbra. In contrast, an induction of TGF-β RIII mRNA was not observed in the intact tissue ((1-way ANOVA for cortex: F = 2.70; for striatum: F = 1.20; Fig. 4C). It was also demonstrated in bright field that TGF-β receptor signals represent labeling of individual cells as autoradiography grains were accumulated above cell bodies (Fig. 3E–H).

Alk1 mRNA was also apparent at 72 h after MCAO. The distribution of induced Alk1 mRNA was similar to that of TGF-β RIII (Fig. 3D). However, the intensity of labeling was more intense for Alk1 (Fig. 3H). Also similar to TGF-β RIII, Alk1 mRNA was not induced in the intact brain tissue (Fig. 4D).

#### 1 month after transient MCAO

**Figure 5:**
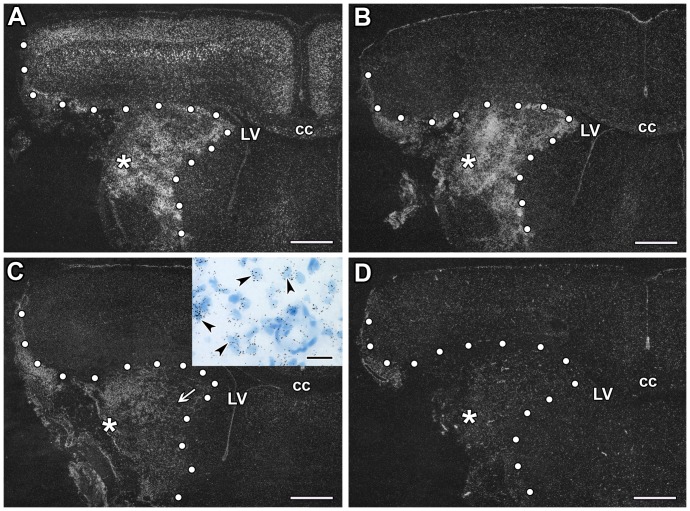
The induction of TGF-β receptors at 1 month following MCAO. The induction of mRNA of TGF-β receptors is demonstrated by dark-field images of sections labeled with in situ hybridization histochemistry. The lesion sites are indicated by star symbols (*) and the borders of lesions are demarcated by white dots. A: TGF-β RI mRNA is abundant within the infarct area. Outside the lesion, TGF-β RI is distributed in the cerebral cortex as in not operated control animals. B: The level of TGF-β RII mRNA is markedly elevated within the lesion. C: TGF-β RIII also appeared in the infarct area. The inlet shows a bright-field image demonstrating the presence of autoradiography grains above non-epithelial cells. D: ALK1 mRNA was slightly elevated within the lesion. Abbreviations: cc - corpus callosum, LV - lateral ventricle. Scale bars: 1 mm for all panels and 20 µm for the inlet in panel C.

TGF-β RI and RII expression remained very high within the infarct area (Fig. 5A, B). In fact, the remaining, not shrunken tissue showed an even higher expression level of TGF-β RI and RII mRNA at 1 mo than at 72 h following MCAO (Fig. 4). Interestingly, TGF-β RIII mRNA was further induced within the lesion and its distribution became similar to that of TGF-β RI and RII (Fig. 5C). In contrast, the distribution and labeling intensity of Alk1 remained similar to that at 72 h (Fig. 5D). A penumbra was not identifiable at this time point. Outside of the lesion, only TGF-β RI showed abundant labeling with a distribution similar to the intact brain in the cerebral cortex but was not induced above normal levels (Fig. 5A).

### The cell types expressing TGF-β receptors

**Figure 6:**
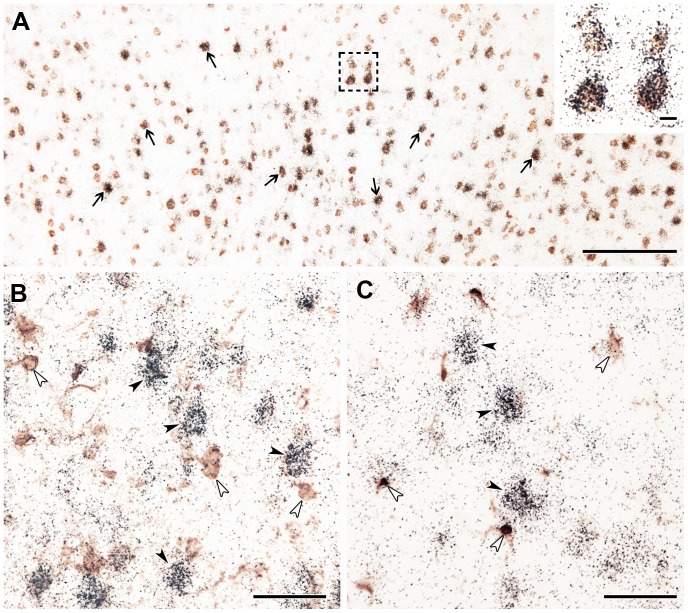
TGF-β RI mRNA expression in neurons of the intact cerebral cortex. TGF-β RI predominantly expressed in neurons and not in glial cell types in the normal cerebral cortex. A: Double labeling of TGF-β RI mRNA and NeuN immunoreactivity. The black in situ hybridization signal co-localizes with brown NeuN immunoreactivity in a large number of cells. The back arrows point at some double labeled cells. The rectangle indicates the position of the high magnification picture in the inlet. It shows the presence of autoradiography grains above NeuN-immunopositive neuronal cells. B: Single labeled TGF-β RI mRNA expressing cells are indicated by black, and S-100 immunoreactive astrocytes by white arrowheads. C: TGF-β RI mRNA expressing neurons are indicated by black, and Iba1-immunoreactive microglias by white arrowheads. There are no double labeled cells present for TGF-β RI and the astrocyte marker S-100 or the microglia marker Iba1. Scale bars  = 200 µm for A, 50 µm for B and C, and 10 µm for the inlet in panel A.

As expected, all markers labeled a large number of cells in the brain outside the lesion. The labeling patterns of the markers were not changed noticeably away from the lesion when compared to the corresponding brain regions in control, not operated animals. The fairly large number of TGF-β RI-expressing cells in the cerebral cortex away from the lesion was neurons and only a few of them were glial cells. Thus, black in situ hybridization signal co-localized with brown NeuN immunoreactivity in a large number of cells (Fig. 6). 51 out of 54 TGF-β RI-expressing cells in a 0.16 mm^2^ area were double labeled with NeuN in layer IV. of the cortex and only few cells were labeled with glial markers (Table 1). Other layers contained less TGF-β RI-expressing cells but the vast majority was still neurons. The much lower number of cells labeled for other TGF-β receptors in the intact brain tissue was astrocytes (labeled with S100) and neurons (labeled with NeuN) for TGF-β RII, and neurons and endothelial cells (labeled with von Willebrand factor) for TGF-β RIII and Alk1.

**Table 1:** Types of TGF-β receptor-expressing cells based on double labeling with cell type markers.

	TGF-β RI			TGF-β RII			TGF-β RIII			Alk1		
	intact	penumbra	lesion	intact	penumbra	lesion	intact	penumbra	lesion	intact	penumbra	lesion
**Number of TGF-β receptor cells/0.16 mm^2^**	57.4±2.6	58.6±2.7	46.1±3.2	8.3±0.8	36.4±2.3	34.3±2.8	11.9±1.2	10.8±0.9	9.9±0.6	6.3±0.9	16.8±1.6	21.3±1.8
**Number of double labeled cells/0.16 mm^2^**												
**NeuN**	50.5±1.9	17.5±2.7	2.0±0.8	2.8±0.5	2.5±1.0	0.5±0.3	5.5±1.2	1.0±0.4	0.3±0.3	3.0±0.4	1.5±0.3	0.0±0.0
**S100**	4.3±0.5	2.5±0.3	0.8±0.5	4.8±0.5	3.5±0.6	0.8±0.3	1.5±0.6	0.5±0.3	0.3±0.3	0.5±0.3	0.5±0.3	0.0±0.0
**Iba1**	2.5±0.3	40.3±1.7	41.3±4.5	1.0±0.4	26.5±1.2	23.0±0.9	0.3±0.3	0.8±0.5	0.8±0.5	0.5±0.3	1.5±0.3	1.5±0.3
**vWF**	0.5±0.3	1.5±0.5	1.5±0.5	1.3±0.6	5.3±0.9	12.5±1.3	6.8±1.5	9.5±0.5	8.3±0.6	3.3±1.0	16.0±1.5	20.8±1.5

Double labeling was performed by in situ hybridization histochemistry for the TGF-β receptors and immunohistochemistry for markers of different cell types at 72 h after MCAO. To identify the cell types the following markers were used: NeuN for neurons, S-100 for astroglial cells, Iba1 for microglial cells, and vWF for endothels. Data on TGF-β receptors and double labeled cells are presented in 3 different locations. The cell counts were performed in 400 µm×400 µm rectangular-shaped areas of the intact (away from the lesion), penumbral and lesioned cerebral cortex from 4 brains.

**Figure 7:**
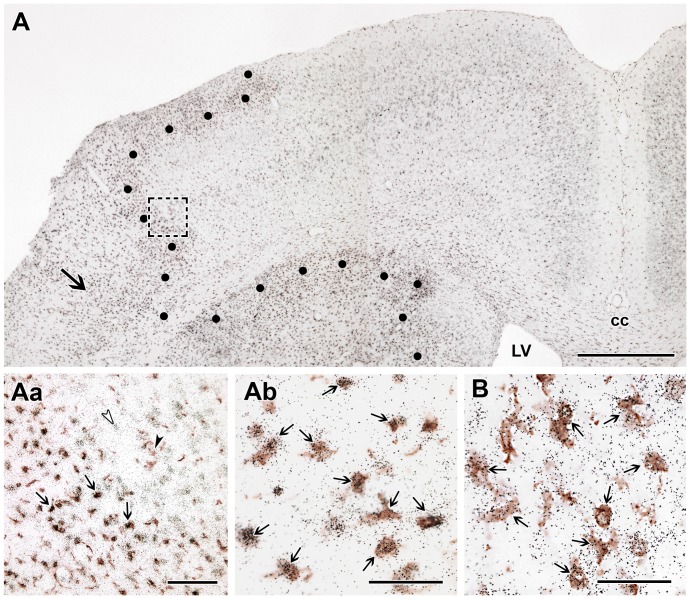
TGF-β RI and RII are induced in microglial cells. Double labeling of TGF-β RI and RII mRNA and immunoreactivity of the microglia marker Iba1 at 72 h following MCAO. A: A low magnification picture of a section double labeled with TGF-β RI and Iba1. The border of the lesion is indicated by black dots. A rectangle and a large black arrow show the position of high magnification pictures in Aa and Ab, respectively. Aa: Single labeled TGF-β RI mRNA expressing neurons are indicated by white, and Iba1-immunoreactive microglia by black arrowheads in the intact cortex away from the lesion, respectively. In the penumbra, double labeled cells are found, some examples are shown by small black arrows. Ab: A high magnification picture shows that the black in situ hybridization signal of TGF-β RI is located above Iba1-imunoreactive cell bodies (brown precipitate) within the lesion. B: TGF-β RII is also expressed in microglia as demonstrated by double labeling of TGF-β RII mRNA (black in situ hybridization signal) and immunoreactivity of Iba1 (brown precipitate). TGF-β RII mRNA expressing microglias are indicated by black arrows. Abbreviations: cc - corpus callosum, LV - lateral ventricle. Scale bars: 1 mm for A, 100 µm for Aa and 50 µm for Ab and B.

**Figure 8:**
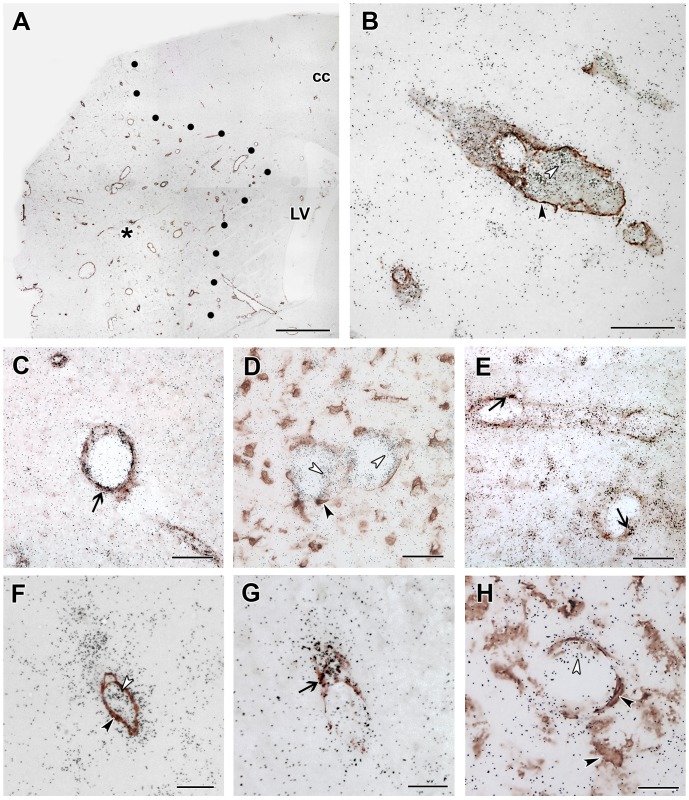
TGF-β RIII and ALK1 are induced in endothelial cells within the lesion. Double labeling of TGF-β RIII and ALK1 mRNA and immunoreactivity of the microglial marker Iba1, the endothelial marker vWF, and the marker of smooth muscle cells of vessels, □SMA at 72 h after MCAO. A: Double labeling of ALK1 mRNA and alpha-SMA immunoreactivity in a low magnification image. The lesion site is indicated by star symbol (*). The border of the lesion is shown by black dots. B: A higher magnification picture of a section double labeled with ALK1 mRNA and alpha-SMA immunoreactivity. Single labeled ALK1 mRNA expressing cells are indicated by white and alpha-SMA-immunoreactive smooth muscle cells by black arrowheads. C: Double labeling of ALK1 mRNA and vWF-immunoreactive endothels. The field indicated by the black arrow shows an example of double labeling. D: ALK1 mRNA (black grains, some cells indicated by white arrowheads) do not co-localize with Iba1 immunoreactivity (brown precipitate, some cells indicated by black arrowheads). E: TGF-β RII mRNA is present in vWF-immunoreactive endothels as well as in some non-endothelial cells in the representative figure. The last 3 panels demonstrate that TGF-β RIII mRNA is expressed in endothelial cells at 72 h after MCAO. F: A double labeling of TGF-β RIII and alpha-SMA shows no co-localization. A single labeled TGF-β RIII mRNA expressing cell is indicated by black, while an alpha-SMA immunoreactive smooth cell by a white arrowhead. G: TGF-β RIII-expressing cells contain vWF immunoreactivity. Double labeled cells are indicated by the black arrow. H: TGF-β RIII mRNA is not present in expressed in microglia. The white arrowhead points to a TGF-β RIII-expressing cell while the black arrowhead shows an Iba1-immunoreactive microglia. Abbreviations: cc - corpus callosum, LV - lateral ventricle. Scale bars: 1 mm for A, 50 µm for B, C, D and E, and 20 µm for F, G and H.

In the brain sections of not operated and sham operated rats, Iba1 immunohistochemistry labeled resting microglia with ramified thin processes. In the perimeter of the lesion, intensely Iba1-ir, large, ameboid-shaped cells were present 24 h after MCAO suggesting the appearance of activated microglia. At 72 h after MCAO, Iba1 immunoreactivity was further increased around the lesion. In addition, Iba1-ir cells were visible within the infarct area (Fig. 7). The distribution of Iba1-ir cells was similar to that of TGF-β RI and RII-expressing cells. Furthermore, a combination of Iba1 immunohistochemistry and *in situ* hybridization for TGF-β RI and RII indicated co-localization of Iba1 and these 2 receptor types within the ischemic core as well as around the lesion (Fig. 7). Almost all TGF-β RI (41 out of 46 within a 0.16 mm^2^ area), and the majority of TGF-β RII-expressing cells 23 out of 34) contained Iba1 while most Iba1-ir cells in and around the lesion expressed TGF-β RI and RII (Table 1). In contrast, the distribution of TGF-β RIII and Alk1-expressing cells had different distributions and showed no co-localization with Iba1 (Fig. 8). Both S100- and NeuN-ir cells were essentially absent in the ischemic core. Thus, apart from the above described Iba1-positive microglia, blood vessels were present within the infarct. In fact, the labeling intensity of immunolabeling with vWF and alpha-SMA was higher within the infarct than outside of it (Fig. 8A). Double labeling with ALK1 mRNA and alpha-SMA immunoreactivity demonstrated that the cells labeled with different signals are in each other's proximity but no double labeling was visible (Fig. 8B). Analysis of double labeled sections at high magnification revealed that both Alk1 (Fig. 8C) and TGF-β RIII (Fig. 8G) are expressed in endothelial cells labeled with vWF but not in smooth muscle cells and microglia (Fig. 8B, D, F, H). For TGF-β RIII, 8 out of 10 cells, while for Alk1, 21 out of 21 cells in a 0.16 mm^2^ area were positive for vWF within the infarct. In addition, TGF-β RII was also expressed in endothelial cells (Fig. 8E), in a ratio of 13 out of 34 cells (Table 1). In contrast, only a low ratio of TGF-β RI-expressing cells was endothels: 2 out of 46 cells were double labeled with vWF (Table 1).

## Discussion

Our findings indicate that TGF-β receptors are induced in the brain following focal ischemic attack and that the mRNAs of different types of TGF-β receptors have individual topographical distributions in distinct cell types. We compare these patterns to the distributions of previously published expressional data of TGF-β receptors following MCAO, and also provide some information on the mechanisms involved in the inductions. Finally, our data are discussed in terms of the potential functions of the TGF-β system in brain ischemia.

### Novel findings on the expression of TGF-β receptors in the brain following focal ischemia and their comparisons with previous results

This study was unique in that it examined the expression and induction of all 4 types of TGF-β receptors. Even though a considerable size of lesion was found 24 h after the MCAO, the change in the level of TGF-β receptors was not significant at this time point. The lack of labeling within the lesion suggests that the degrading neurons do not express TGF-β receptors and TGF-β signaling may not be changed in the penumbral tissue, either. In the intact tissue, the level of TGF-β RI was the highest, and it was the only one organized topographically with particularly abundant expression in layer IV. of the cerebral cortex, which implies neuronal localization. It was indeed confirmed by double labeling with NeuN. Neither the pattern of expression of TGF-β RI nor its expression in neurons of layer IV has been reported previously [17]. Most of the other cells labeled with TGF-β RI were astrocytes double labeled with S100. The presence of TGF-β RII in astrocytes was first demonstrated by double labeling, however, it is consistent with previous reports using cultured astrocytes [31]. In contrast, TGF-β RIII and Alk1 were both located in some neurons and vWF-labeled endothels. As for TGF-β RIII, a neuronal localization has been suggested before [35] while Alk1 has been described both in neurons and endothels [36], [38], [41] in concentrations higher than in astrocytes [38].

At 72h and 1 month following MCAO, all types of TGF-β receptors showed significant induction. The spatial and temporal pattern of induction was very similar for TGF-β RI and RII suggesting their co-expression, which is consistent with the functional receptor being a heteromer [4]. Both receptors were markedly induced around the infarct area and within the lesion but not outside the lesion and contralateral to the lesion. The region around the infarct area may correspond to the ischemic penumbra, a major target of neuroprotective treatment [42]. These findings are in agreement with a previous report that TGF-β RII mRNA levels, identified by RT-PCR, are dramatically elevated in the ischemic area 3 days following permanent MCAO in mice [33]. However, our results contrast the same report as an increase was not detected for TGF-β RI in that study [33] as we described the induction of TGF-β RI following MCAO in the penumbra. We believe that the high level of basal expression of TGF-β RI we found in the intact tissue by in situ hybridization histochemistry masked the RT-PCR data. The presented data also contradict a previous immunohistochemical study, which reported that TGF-β RI and RII are induced in neurons, glial cells, and endothels both the ipsi- and contralateral hemisphere in response to MCAO [32]. Although different patterns are conceivable for mRNA and protein induction, we believe that the antibody specificities caused the differences as immunohistochemistry with single antibodies against each subtype tested without using knock-out animals is less reliable than the results obtained by two non-overlapping in situ hybridization probes. Therefore, our study, which first explored the activation of TGF-β receptors following MCAO by in situ hybridization, can contribute to the clarification of contradictory findings.

The majority of the cells that we identified with elevated levels of TGF-β RI and RII were microglia, which we first confirmed by double labeling using Iba1, an established marker of both ramified and activated microglia [43]. This finding is in accordance with a previous report describing that global ischemia evoked by a single hypoxic exposure led to a concomitant upregulation of TGF-β RI and RII mRNA and protein measured by RT-PCR and Western blot, respectively [34]. Furthermore, immunohistochemistry showed that TGF-β RI and RII expression occurred in microglia (identified by a lectin of *Lycopersicon esculentum* that label both microglia and blood vessel endothelial cells) and microglial cell cultures were also shown to elevate their expression of TGF-β RI and RII [34]. The present study using in situ hybridization histochemistry and a marker specific to microglia established that focal ischemia also leads to the microglial induction of TGF-β RI and RII. Utilizing the superior spatial resolution of in situ hybridization over RT-PCR and Western blot techniques, we established that the expression level of TGF-β RI and RII is even higher in the penumbra than within the lesion at 72 h after MCAO. It was also first demonstrated that the microglial expression of TGF-β RI and RII is further increased within the infarct by 1 month after MCAO.

TGF-β RIII mRNA level showed a much smaller increase in the penumbra and within the lesion at 72 h after MCAO than TGF-β RI and II. Furthermore, the cells that exhibited elevated expression level of TGF-β RIII at this time point were predominantly endothels, a result is first reported in the current study. Since endothels were found to contain only minimal amount of TGF-β RI, it is possible that TGF-β RIII may mediate other ligands that TGF-βs including bone morphogenetic proteins (BMPs) [14], [44], [45]. The lack of TGF-β RIII in microglia at 72 h following MCAO, a finding that has not been previously reported, suggests that TGF-β RI and RII transmit TGF-β action without TGF-β RIII receptors at this time point. According to previous data, TGF-β RI and RII can indeed function in the absence of TGF-β RIII as TGF-β receptor [46] except that the recognition of TGF-β2 is missing [9]. However, we also first established that TGF-β RIII appears in non-endothel cells within the lesion at 1 month following MCAO suggesting that TGF-β RI and RII can recognize all TGF-β subtypes at this time point.

Alk1 expression showed a significant increase at 72 h after MCAO in the penumbra and lesion. However, unlike TGF-β RIII, its level has not been further elevated by 1 month after MCAO. These findings are entirely novel and consistent with previous results showing that Alk1 is induced in preexisting feeding arteries and newly forming vessels during wound healing [41] and that it becomes strongly upregulated in endothelial cells of following endothelial denudation in vitro and in vivo [37]. Our results first demonstrated that the elevation of Alk1 expression takes place in endothelial cells following MCAO and we also showed that TGF-β RII is also induced in endothelial cells. However, we did not observe elevated Alk1 mRNA levels in neurons, which is different from previous immunohistochemical results [38]; it possible that an elevation in protein levels is not reflected at the mRNA level. As a type I receptor, Alk1 may require a type II receptor for signal transduction. Indeed, an elevated level of TGF-β RII is present in endothelial cells allowing the recognition of TGF-β [4], [14]. In addition, Alk1 may also bind to other type II receptors to recognize ligands others than TGF-βs, such as BMP-9 and -10 [47]. Another interesting and novel point of our results is the similarity in distribution between mRNA of Alk1 and TGF-β RIII allowing potential interactions between these receptors. Thus, the present study largely expanded our knowledge on the induction of TGF-ß receptors following MCAO as it first described the expression of 4 types of TGF-β receptors simultaneously at different time points following an ischemic attack.

### Possible mechanisms of induction of TGF-ßs

The spatial and temporal patterns of induction was the same in the area of the cerebral cortex and the caudate putamen (striatum) suggesting that the mechanisms of induction of TGF-ß receptors does not depend on the structure of the surrounding brain tissue. Since the expression level of TGF-ß receptors was not different between permanent occlusion and 1 h occlusion followed by reperfusion, it is likely that ischemic damage itself rather than reperfusion evokes the induction of TGF-ßs. Focal ischemic damage is known to activate microglia in the infarct area as well as in the adjacent surviving area [48] Microglial activation predominates over macrophage infiltration following MCAO [49]. Since the expression levels were significantly higher in the penumbra than within the lesion at 72 h, we suggest that the elevated TGF-β RI and RII are in activated microglia invading the infarct and that the increased expression of TGF-β RI and RII is predominantly a consequence of microglial activation. Inflammatory cytokines released, e.g. tumor necrosis factor-alpha and IL-1 have been shown to induce TGF-ß1 expression in microglia and astrocytes [50], [51]. Cytokines, including TGF-βs themselves contribute to the induction of TGF-β receptor levels. TGF-βs are released in response to hypoxia [34], [52], which has been shown to induce TGF-β RI and RII [52], [53]. Interestingly, TGF-β RIII expression is negatively regulated by TGF-β1 at least in some cancer cells [54] and testicular cells [55]. This represents a possible mechanism why TGF-β RIII is not expressed at 72 h in microglial cells. In contrast to TGF-β RI and RII, TGF-β treatment does not increase Alk1 expression [38], which may in turn be upregulated in endothelial cells by soluble cytokines, e.g. interleukin 6, released by injury [37].

### The TGF-β system following MCAO and its potential functions in focal ischemia

**Figure 9:**
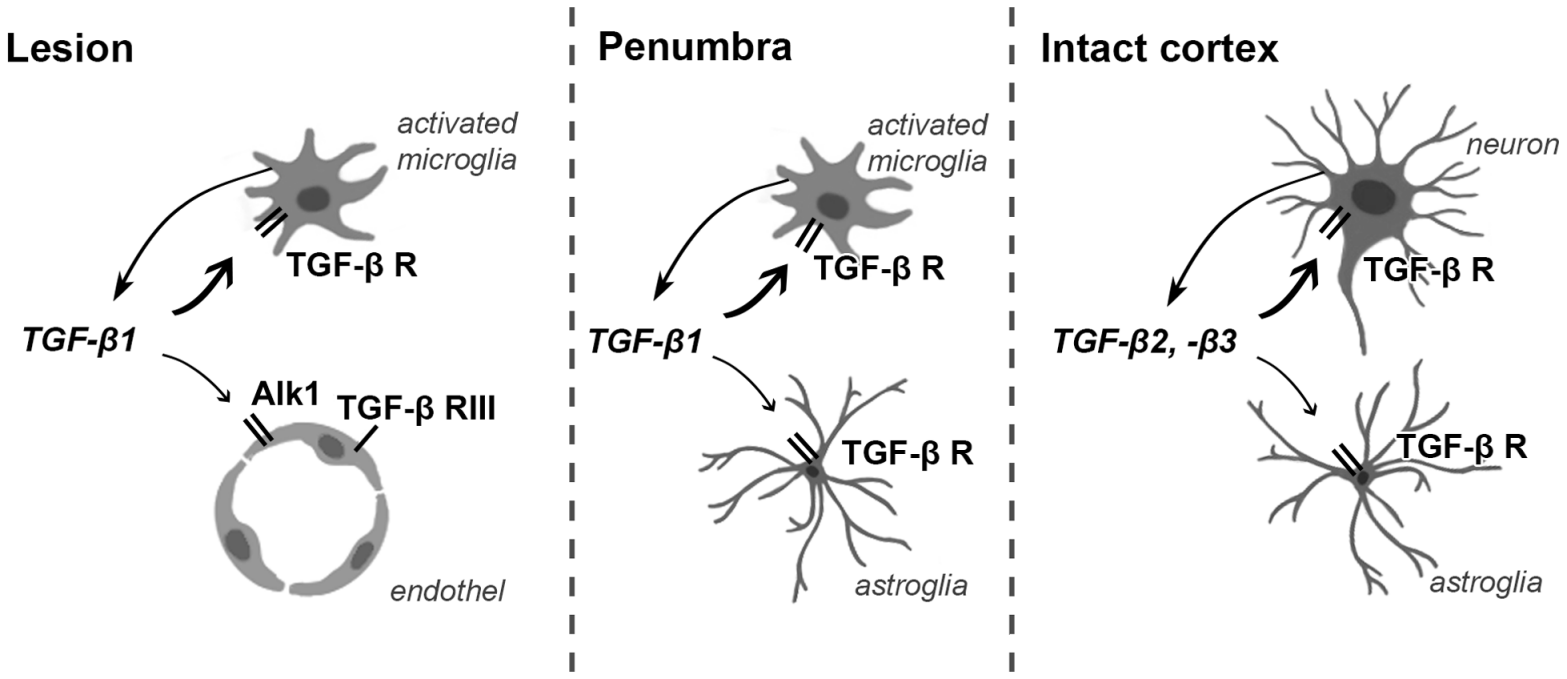
Schematic figure on TGF-β signaling at 72 h after MCAO. In the infarct area, TGF-β1 released from microglial cells acts on microglial and endothelial cells via TGF-β RI and Alk1, respectively. In the penumbra, TGF-β1 released from astrocytes and microglial cells act on these glial cells through TGF-β RI. Meanwhile, in the intact tissue away from the lesion, TGF-β2 and -β3 are released from neurons act on neurons and astrocytes by means of TGF-β RI.

Following MCAO, le TGF-β1 is induced in periinfarct areas all around the lesion, while TGF-β2 and -β3 are expressed in specific cortical layers (layers II., IV., V.) not only in the penumbra but also in remote regions of the ipsilateral hemisphere by 24 h following MCAO [20]. Thus, the activation of TGF-βs precedes that of TGF-β receptors. At 72 h and 1 month after MCAO, the distribution of TGF-β1 is very similar to that of TGF-β receptors. TGF-β1 is expressed in the penumbra as well as within the lesion in microglial and to a lesser extent in astroglial cells [20]. At 1 month after MCAO, TGF-β2 also appears in the infarct area. Thus, TGF-β1 released from glial cells is the ligand, which activates TGF-β receptors within the infarct (Fig. 9). In turn, at 1 month after MCAO, TGF-β2 may also contribute to it. TGF-β1 may act on microglial cells containing only TGF-β RI and RII at 72 h following MCAO while at 1 month, TGF-β RIII may also be involved. TGF-β RIII is required for the action of TGF-β2 [44] but not that of TGF-β1 [1], [46], which is consistent with the concomitant appearance of TGF-β2 and TGF-β RIII. The released TGF-βs can also directly influence endothel cells by acting on Alk1 receptors (Fig. 9). Meanwhile, TGF-β2 and -β3, induced in neurons away from the lesion in the ipsilateral cortex [20], may act on TGF-β receptors we found there (Fig. 9).

TGF-βs have neuroprotective actions against hypoxic events at least partially mediated by microglial cells [24], [25], [27], [56]. TGF-β strongly enhances IL4-induced alternative activation of microglia, which is in turn impaired after blocking the TGF-β RI [57]. This alternative activation promotes tissue repair and extracellular matrix reconstruction [58]. At the same time, TGF-β blocks the classical activation of microglia induced by Th1 cytokines [59], [60], which represents a negative auto-feedback inhibitory action on microglial function required to remove debris but also contributing to neuronal damage [61]. TGF-βs are not only capable of deactivating microglia [59] but even promote its selective apoptosis thereby reducing inflammation-mediated neurotoxicity [62], [63].

TGF-βs acting on endothelial cells may also participate in other processes following ischemia including neoangiogenesis [64], [65]. Alk1 receptor was shown to inhibit angiogenesis using neutralizing antibodies against Alk1 [47], an effect independent of vascular endothelial growth factor signaling [66]. Furthermore, increasing the expression of Alk1 in cultured endothelial cells enhanced the TGF-β/Alk1 signaling pathway and endothelial cell functions like tubulogenesis and migration [67]. Based on these functions of the Alk1 receptor, it may also mediate the angiogenesis-promoting effect of TGF-βs following MCAO.

TGF-β receptors on glial cells may in turn be involved in the regulation of glial scar formation [21]. Local injection of a TGF-β antagonist into cerebral wounds reduced glial scarring [68] and abolished the fibrinogen-induced effects on glial scar formation [69] likely by affecting the proliferation, migration and activation of astrocytes [17], [70] and the extracellular matrix environment [71].

Direct actions on neurons have also been implicated in the neuroprotective functions of TGF-βs [56], [72], [73]. Cortical neurons are known to exhibit programmed cell death following MCAO [74]–[77]. Since TGF-β2 and -β3 inhibit hypoxia-induced neuronal apoptosis [56], [72], and TGF-β2 and -β3 are induced in the cortex [20], theymay exert neuroprotective functions by inhibiting apoptosis in the ipsilateral cerebral cortex. In addition to neuronal survival, TGF-βs might also be involved in neuronal repair, a process increasingly investigated for post-stroke intervention [78]. After ischemic lesions, the restoration of neural functions requires novel neurite growth and synapse formation, processes that have also been shown to be influenced by TGF-βs [79]–[81] while ischemia-induced neural stem cell proliferation and differentiation may also include TGF-β pathways [22], [23].

## Conclusions

The induction TGF-β receptors have distinct spatial and temporal resolutions suggesting their involvement in different functions. In the penumbra and within the infarct area, there is a profound induction of TGF-β RI and RII in microglial cells suggesting the effects of TGF-β1 and at later time points by TGF-β2 in the regulation of microglial function. In turn, Alk1 is induced in epithelial cells within the infarcts, which implies the role of this receptor type in angiogenesis followed by MCAO. TGF-β RIII is expressed mainly in endothel cells at early time points but also in other cells within the infarct by 1 month after MCAO. TGF-β receptors are also present in astrocytes and neurons in the penumbra, and the intact cortex outside of the lesion where they may be involved in the regulation of glial scar formation, and anti-apoptotic/neuronal repair functions, respectively. The alterations in TGF-β receptor expression following MCAO suggests that similar changes take place in and around the lesions in stroke patients, too.

## Supporting Information

Figure S1 **The expression of mRNA of TGF-β receptors in the intact brain.** Dark-field photomicrographs of in situ hybridization histochemistry sections are shown contralateral to the lesion (a, left column, 24 h following MCAO), ipsilateral to the sham operation (b, middle column, 24 h following sham operation), and in control brains without surgery (c, right column). There are no differences between the expression of TGF-β RI (A) at the side contralateral of the lesion (Aa), ipsilateral to the sham operation (Ab) and in control (Ac) brains. Likewise, the expression of TGF-β RII (B), TGF-β RIII (C), and ALK1 (D) mRNA are the same in these types of intact brain tissues, which means very low level of expression in all these cases. Abbreviations: cx – cerebral cortex, cp – caudate putamen. Scale bar  = 1 mm.
(TIF)

Figure S2 **Nissl staining of the sham operated and the lesioned, freshly dissected brains at different time points after MCAO.** The first column (a) shows sections from the sham operated rats at 24 hours following transient (Aa) and permanent (Ba) MCAO, at 72 hours (Ca), and 1 month after transient MCAO (Da). The Nissl labeling are the same at the different time points in sham operated rats without any sign of tissue damage. The other columns demonstrate the lesioned brain area in 6 animals per time points at 24 hours after transient MCAO (A1, A2, A3, A4, A5 and A6), at 24 hours after permanent MCAO (B1, B2, B3, B4, B5 and B6), at 72 hours after transient MCAO (C1, C2, C3, C4, C5 and C6) and 1 month after transient MCAO (D1, D2, D3, D4, D5 and D6). The borders of lesions are demarcated by red dots in the Nissl sections. Scale bar  = 1 mm.
(TIF)
