# Supplementary figures and images for: Induction of Transforming Growth Factor Beta Receptors following Focal Ischemia in the Rat Brain
Source: PLoS One. 2014 Sep 5;9(9):e106544. doi: 10.1371/journal.pone.0106544 (PMC4156357; doi:10.1371/journal.pone.0106544)

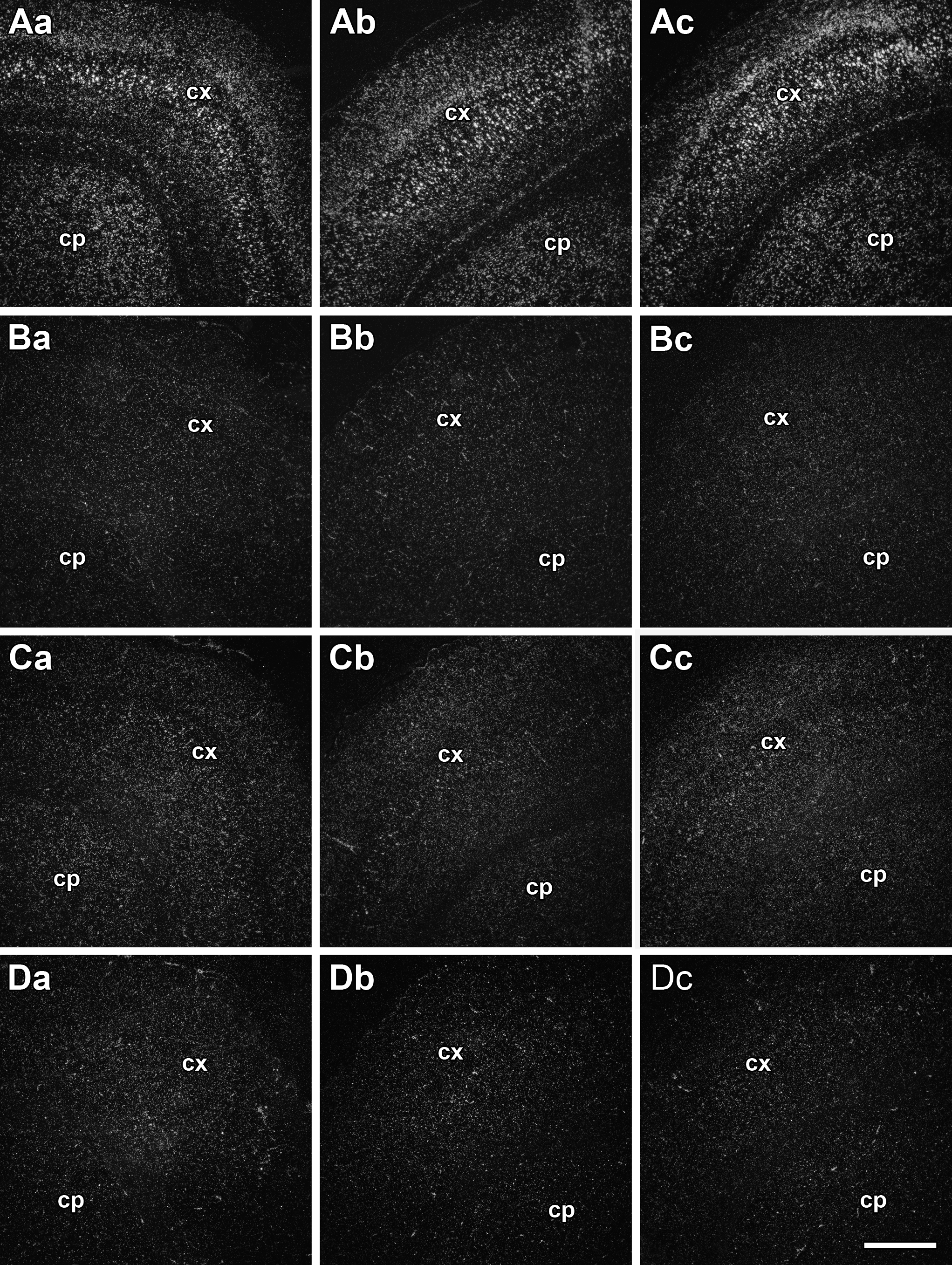

Supplement: Figure S1 — The expression of mRNA of TGF-β receptors in the intact brain. Dark-field photomicrographs of in situ hybridization histochemistry sections are shown contralateral to the lesion (a, left column, 24 h following MCAO), ipsilateral to the sham operation (b, middle column, 24 h following sham operation), and in control brains without surgery (c, right column). There are no differences between the expression of TGF-β RI (A) at the side contralateral of the lesion (Aa), ipsilateral to the sham operation (Ab) and in control (Ac) brains. Likewise, the expression of TGF-β RII (B), TGF-β RIII (C), and ALK1 (D) mRNA are the same in these types of intact brain tissues, which means very low level of expression in all these cases. Abbreviations: cx – cerebral cortex, cp – caudate putamen. Scale bar = 1 mm. (TIF) [file pone.0106544.s001.tif]

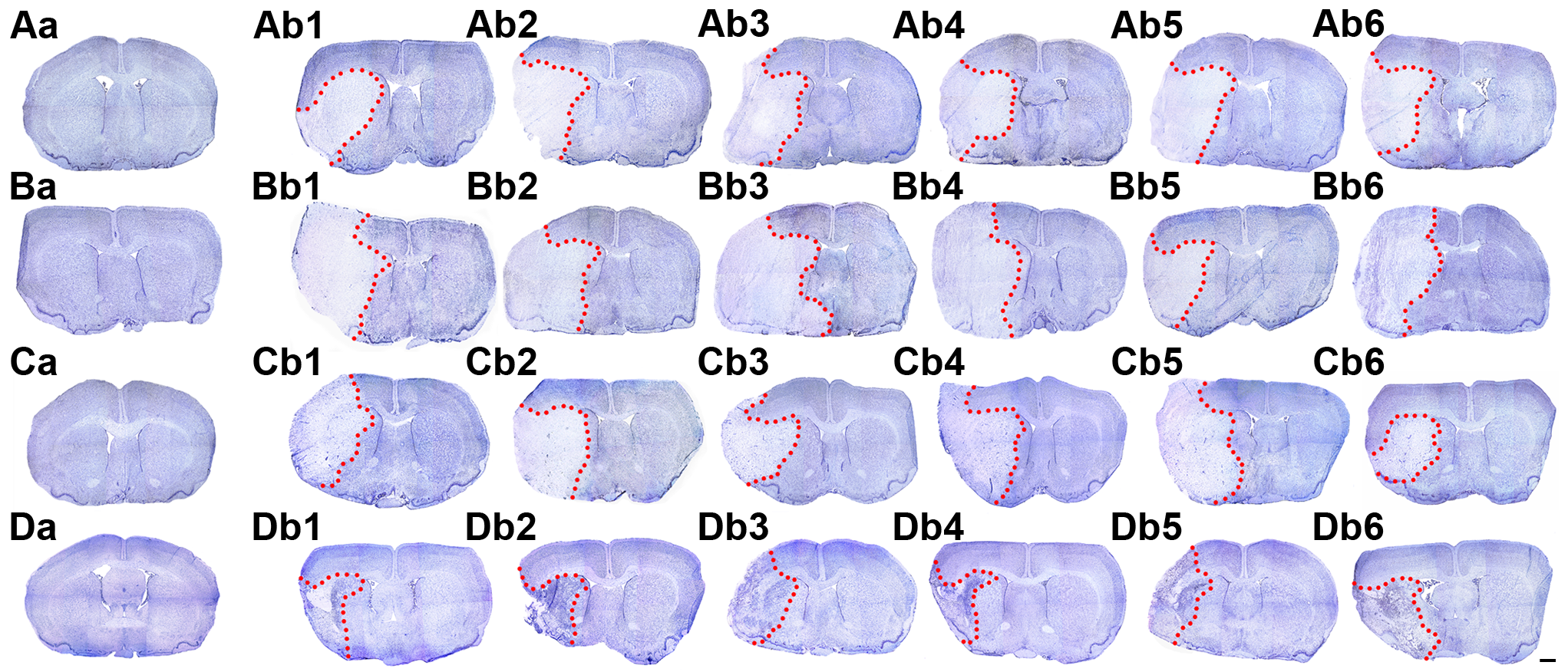

Supplement: Figure S2 — Nissl staining of the sham operated and the lesioned, freshly dissected brains at different time points after MCAO. The first column (a) shows sections from the sham operated rats at 24 hours following transient (Aa) and permanent (Ba) MCAO, at 72 hours (Ca), and 1 month after transient MCAO (Da). The Nissl labeling are the same at the different time points in sham operated rats without any sign of tissue damage. The other columns demonstrate the lesioned brain area in 6 animals per time points at 24 hours after transient MCAO (A1, A2, A3, A4, A5 and A6), at 24 hours after permanent MCAO (B1, B2, B3, B4, B5 and B6), at 72 hours after transient MCAO (C1, C2, C3, C4, C5 and C6) and 1 month after transient MCAO (D1, D2, D3, D4, D5 and D6). The borders of lesions are demarcated by red dots in the Nissl sections. Scale bar = 1 mm. (TIF) [file pone.0106544.s002.tif]
